# Establishing Primary Cultures of Adult Mouse Cardiac Fibroblasts: A Comparative Evaluation of Three Experimental Protocols

**DOI:** 10.3390/ijms27156630

**Published:** 2026-07-25

**Authors:** Evelyn-Gabriela Nastase-Rusu, Ana-Mihaela Lupan, Mihai Bogdan Preda, Alexandrina Burlacu

**Affiliations:** 1 Institute of Cellular Biology and Pathology “Nicolae Simionescu”of the Romanian Academy, 050568 Bucharest, Romania; evelyn-gabriela.rusu@icbp.ro (E.-G.N.-R.); bogdan.preda@icbp.ro (M.B.P.); 2 Department of Cell Biology, Harvard Medical School, Boston, MA 02115, USA; 3 Genomics Research and Development Institute, 020021 Bucharest, Romania

**Keywords:** cardiac fibroblasts, adult mouse heart, cell isolation, primary culture, enzymatic digestion, collagenase, Liberase, flow cytometry, cardiac cells

## Abstract

Cardiac fibroblasts (cFbs) are important in vitro models for studying myocardial fibrosis, extracellular matrix homeostasis, and tissue remodeling. However, establishing primary cultures from adult mouse hearts remains technically demanding due to the limited proliferative capacity of freshly isolated cells, their susceptibility to enzymatic and mechanical stress during tissue dissociation, and the resulting difficulty in obtaining sufficient numbers of viable fibroblasts for downstream experiments. Even small variations in digestion and early culture conditions can affect cell recovery, purity, and expansion, highlighting the need for optimized and reproducible isolation protocols. Here, we compared three enzymatic strategies for establishing primary adult mouse cardiac fibroblast cultures: sequential digestion with collagenase I and trypsin, retrograde coronary perfusion with collagenase II, and continuous digestion with Liberase DH. The three protocols were evaluated by assessing post-isolation cell viability, the composition of recovered non-myocyte populations by flow cytometry, and the ability of isolated cells to establish and expand during the first five days of culture. Retrograde coronary perfusion generated viable fibroblasts but yielded relatively few cells and is most advantageous when fibroblasts are isolated simultaneously with cardiomyocytes from the same heart. Liberase DH digestion recovered a broader spectrum of non-myocyte populations, making it suitable for immediate phenotypic analyses, although it produced less efficient fibroblast expansion in culture. Sequential collagenase I/trypsin digestion showed the best overall performance, with low mortality, negligible CD31^+^ endothelial contamination, and robust expansion into confluent spindle-shaped monolayers within five days. Overall, these findings identify sequential collagenase I/trypsin digestion as an effective and reproducible approach for establishing viable adult mouse cardiac fibroblast cultures for downstream functional and molecular studies.

## 1. Introduction

The adult mammalian heart contains a complex interstitial compartment composed of fibroblasts, endothelial cells, immune cells, smooth muscle cells, pericytes, and other cell types. Among them, cardiac fibroblasts constitute the major stromal cell compartment essential for extracellular matrix (ECM) synthesis and turnover, paracrine signaling, and the structural response to cardiac stress [1,2,3].

Accordingly, primary cultures of adult cardiac fibroblasts are indispensable experimental models for investigating the molecular and cellular mechanisms of myocardial fibrosis, extracellular matrix remodeling, and cardiac repair [3].

However, obtaining sufficient numbers of viable fibroblasts that retain their capacity to establish and expand in primary culture remains technically demanding. The myocardium is dense, collagen-rich, and structurally heterogeneous, whereas fibroblasts lack a single universal surface marker that unequivocally identifies all subsets [4]. An optimal isolation protocol should efficiently dissociate the adult myocardium, preserve cell viability, enrich for cardiac fibroblasts while minimizing endothelial and hematopoietic cell carryover, and generate cultures capable of robust expansion with minimal spontaneous activation. Several approaches have been developed to isolate adult cardiac fibroblasts or broader non-myocyte populations, including direct tissue digestion, explant-based culture, retrograde coronary perfusion, and continuous enzymatic digestion using Liberase [5,6,7,8]. These approaches differ in technical complexity, digestion intensity, cell yield, and suitability for downstream applications. For example, explant-based culture protocols are simple and accessible but initially yield heterogeneous non-myocyte populations. In contrast, perfusion-based protocols are advantageous when cardiomyocytes and non-myocytes are isolated simultaneously from the same heart [8,9].

Here, we compared three cardiac fibroblast isolation strategies: sequential enzymatic digestion with collagenase I and trypsin, retrograde coronary perfusion with collagenase II, and continuous digestion with Liberase DH. The rationale for selecting these three isolation protocols was based on their distinct experimental objectives and their representation of different strategies for cardiac fibroblast isolation. The first protocol was specifically optimized for the isolation of cardiac fibroblasts, without the intention of recovering other cardiac cell populations. In contrast, the second protocol was originally designed for the isolation of cardiomyocytes while also allowing the recovery of cardiac fibroblasts as a by-product of the isolation procedure. The third protocol was included to evaluate the impact of a rapid enzymatic digestion strategy on fibroblast yield and quality. Together, these protocols represent three conceptually different approaches to cardiac fibroblast isolation, allowing us to compare methods that differ in their primary purpose, digestion strategy, and expected cellular outcome. The aim was to identify the method that most effectively generates viable, fibroblast-enriched primary cultures from adult mouse hearts that can attach, expand, and support downstream in vitro studies, while also clarifying when alternative protocols may be preferable for specific experimental objectives.

## 2. Results

### 2.1. Sequential Digestion Produced the Highest Cardiac Fibroblast Yield

To establish a reliable primary cardiac fibroblast culture suitable for downstream in vitro studies, we compared three isolation strategies designed to recover viable cardiac interstitial cells from adult mouse hearts. The protocols differed in enzyme buffer composition and mechanical dissociation strategy: Protocol 1 (P1) was based on sequential digestion with collagenase I and trypsin; Protocol 2 (P2) used retrograde coronary perfusion with collagenase II followed by recovery of the non-myocyte fraction; and Protocol 3 (P3) relied on continuous enzymatic digestion with Liberase DH. Immediately after isolation, cell viability was assessed by PI-based flow cytometry.

**Figure 1:**
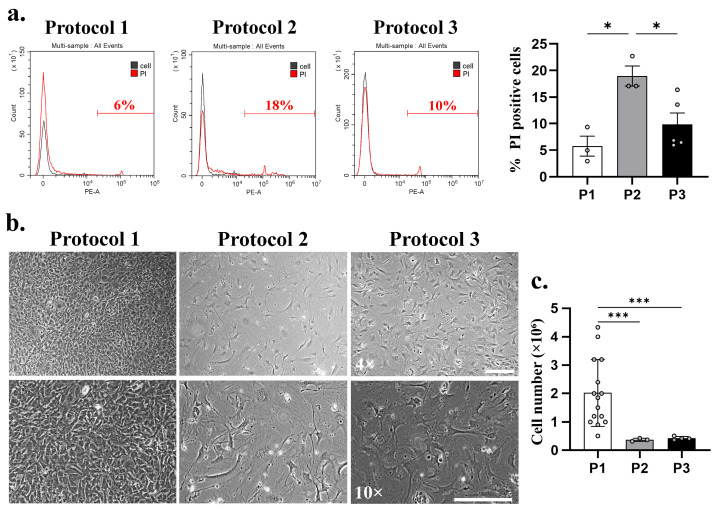
Comparative assessment of cardiac fibroblast isolation protocols. (**a**) Left: Representative flow cytometry histograms showing propidium iodide (PI) staining immediately after isolation for Protocol 1, Protocol 2, and Protocol 3. Right: Quantification of the percentage of PI-positive cells recovered after each isolation procedure. Protocol 1 showed the lowest cell mortality (6%), whereas Protocol 2 and Protocol 3 showed higher proportions of PI-positive cells (18% and 10%, respectively). Data are presented as mean ± SD; *n* = 3–5 animals per group. Statistical analysis was performed using one-way ANOVA followed by Tukey’s multiple-comparisons test. * *p* < 0.05. (**b**) Representative phase-contrast images of adherent cells after 5 days in culture, showing differences in cell density and morphology between the three protocols. Protocol 1 generated a dense, overconfluent monolayer of spindle-shaped fibroblast-like cells, whereas Protocols 2 and 3 produced lower-density cultures with more heterogeneous cellular morphology. Images are shown at 4× and 10× magnification. Scale bar = 250 µm. (**c**) Quantification of total cell number obtained after 5 days in culture for each protocol. Protocol 1 yielded the highest number of adherent cells compared with Protocols 2 and 3. Data are presented as mean ± SD; *n* = 3–15/animals. Because equal variances were not assumed, differences among groups were analyzed using Welch’s ANOVA followed by Dunnett’s T3 multiple-comparisons test. *** *p* < 0.001.

The results showed clear differences between the three approaches. P1 yielded the lowest proportion of PI-positive non-viable cells, with approximately 6% cell mortality, suggesting that sequential collagenase I/trypsin digestion better preserved cell membrane integrity during tissue dissociation. This favorable viability profile may be explained by the stepwise nature of the protocol, in which cell-containing fractions were collected progressively and rapidly neutralized with FBS, thereby reducing the enzymatic exposure (Figure 1a). In contrast, P2 resulted in the highest mortality rate, with 18% PI-positive cells, which was significantly higher than that observed with P1 and P3 (Figure 1a), most likely reflecting the more intensive enzymatic exposure required during retrograde coronary perfusion, together with the additional mechanical and processing steps associated with cardiomyocyte sedimentation and recovery of the non-myocyte fraction. Protocol 3 showed an intermediate viability profile, with 10% non-viable cells, consistent with efficient but continuous Liberase DH-mediated tissue dissociation, which may increase membrane stress compared with the more gradual digestion used in P1 (Figure 1a).

Next, we assessed the capacity of the isolated cells to adhere and expand in culture over five days. Phase-contrast images showed that P1 generated a uniform and overconfluent culture composed of spindle-shaped cells with high proliferative capacity (Figure 1b). In contrast, P2 and P3 produced cultures with reduced cell density and cells displaying hypertrophic or activated morphology, indicating limited proliferation (Figure 1b). Moreover, Protocol 1 produced the highest cell yield before first passage, with a mean of approximately 2 × 10^6^ cells per heart, which was significantly higher than the yields obtained with Protocols 2 and 3. P2 and P3 yielded markedly fewer cells, with means of approximately 2.5 × 10^5^ and 4 × 10^5^ cells per heart, respectively (Figure 1c).

### 2.2. Sequential Digestion Protocol Emerges as the Optimal Strategy for Cardiac Fibroblast Isolation and Culture

We next assessed the phenotype of the freshly isolated cell fractions by flow cytometry, evaluating *Ly6a*/Sca-1, CD31/*PECAM1*, and CD45/*PTPRC* expression to characterize a heterogeneous Sca-1-positive non-myocyte population, endothelial cells, and hematopoietic cells, respectively. The results showed that the cellular composition differed markedly among the three isolation protocols.

**Figure 2:**
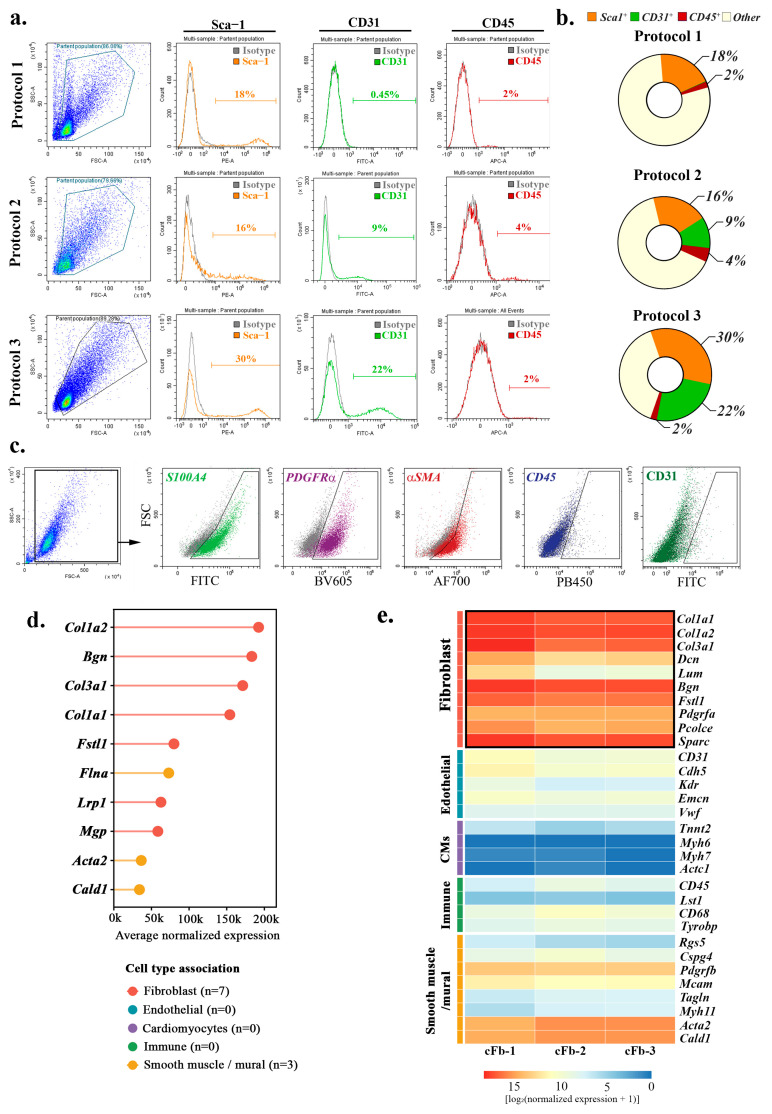
Immunophenotypic characterization of freshly isolated cardiac cell populations and transcriptomic profiling of cultured cells. (**a**) Representative flow cytometry histograms from one independent experiment showing Sca1, CD31, and CD45 expression for each isolation protocol. Isotype controls are shown in grey. The parent cell population was defined by FSC-A/SSC-A gating. (**b**) Donut charts summarizing the relative distribution of Sca-1^+^, CD31^+^, CD45^+^, and other cells within the freshly isolated cell fractions generated by each protocol. Percentages indicate the mean proportion of Sca-1^+^, CD31^+^, and CD45^+^ cells within the gated parent population, calculated from at least three independent experiments. (**c**) Representative flow cytometry dot plots showing the extended immunophenotypic characterization of primary cardiac fibroblasts at passage 0. Cells were analyzed for fibroblast-associated markers S100A4, PDGFRα, and α-SMA, along with CD45 and CD31, to assess residual contamination by hematopoietic and endothelial cells. (**d**) Lollipop plot of the top 10 most highly expressed genes in the bulk RNA-sequencing dataset, ranked by average normalized expression and colored by assigned cardiac cell-type association based on marker genes from the Tabula Muris Senis atlas. (**e**) Heatmap showing the expression of a canonical cell-type-specific marker panel across the three cultured cardiac fibroblast samples grouped by cell-type category (fibroblast, endothelial, cardiomyocytes (CMs), immune, and smooth muscle/mural). Bulk RNA-sequencing counts were normalized using DESeq2 and are shown as log2 (normalized expression + 1).

Liberase DH-based digestion (Protocol 3) generated the broadest non-myocyte recovery, yielding the highest proportions of both Sca-1^+^ cells and CD31^+^ endothelial cells. Quantitative analysis showed that the percentages of Sca-1^+^ and CD31^+^ cells were significantly higher with P3 than with P1 and P2. In contrast, the proportion of CD45^+^ cells remained low and did not differ significantly among the three protocols (Figure S2). This indicates efficient dissociation of the cardiac interstitium, but also substantial recovery of the vascular compartment. In contrast, the collagenase-based protocols (P1 and P2) produced lower percentages of Sca-1^+^ cells (Figure 2a,b). Importantly, Protocol 1 did not yield detectable CD31^+^ endothelial cells or CD45^+^ hematopoietic cells, suggesting a more fibroblast-enriched isolation profile. Consistent with this observation, quantitative analysis showed that Protocol 1 contained significantly fewer Sca-1^+^ and CD31^+^ cells than Protocol 3, further supporting its more selective isolation profile (Figure S2). This represents a major advantage for establishing primary cardiac fibroblast cultures, as contamination with endothelial or hematopoietic cells may affect culture purity and cellular behavior (Figure 2a,b). This finding was further supported by flow cytometric analysis of passage 0 cultures after 5 days in vitro. At this stage, the adherent cell population displayed a fibroblast-enriched phenotype, with detectable expression of fibroblast-associated markers, including S100A4, PDGFRα, and α-SMA. In parallel, the cells remained negative for CD45 and CD31, indicating minimal residual hematopoietic or endothelial contamination (Figure 2c). To further characterize the cellular identity of the cultures using an independent approach, bulk RNA-sequencing data were analyzed against cell-type-associated marker genes from the Tabula Muris Senis mouse heart single-cell RNA-sequencing dataset [10]. Fibroblast-associated genes dominated the top 10 most highly expressed cell-type-assigned genes (7 of 10), with the remaining 3 genes classified as smooth muscle/mural-associated; no genes were assigned to endothelial, cardiomyocyte, or immune populations (Figure 2d). To confirm marker specificity, we selected a panel of cell-type-specific genes. Across all three biological samples, cardiac fibroblast markers showed strong and reproducible expression, while endothelial, immune, and cardiomyocyte markers remained comparatively low (Figure 2e). A limited number of smooth muscle/mural-associated genes (Pdgfrb, Acta2, and Cald1) were also detected, which may reflect fibroblast activation in culture. However, the presence of minor residual mesenchymal populations cannot be completely excluded (Figure 2e).

Together, these data confirm that Protocol 1 efficiently supports the establishment of highly enriched primary cardiac fibroblast cultures suitable for downstream in vitro analyses. Therefore, Protocol 1 was selected for subsequent in vitro characterization and culture optimization.

### 2.3. Middle Digestion Fractions Contributed Most to Protocol 1 Yield

**Figure 3:**
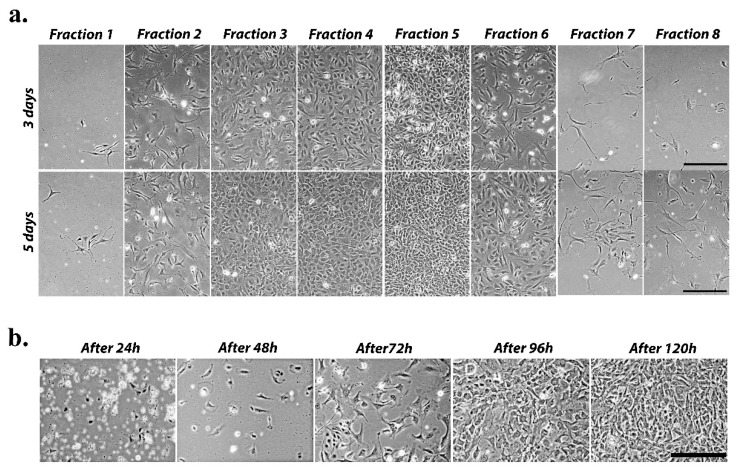
In vitro evolution of cardiac cells isolated by enzymatic digestion into successive fractions. (**a**) Culture appearance on days 3 and 5 for each fraction obtained after digestion of a murine heart. Each fraction was seeded into one well of a 6-well plate. Scale bar: 250 µm; (**b**) representative images of non-myocyte cell cultures isolated through sequential fractions and seeded together in a 10 mm diameter plate, captured over five consecutive days. Scale bar: 250 µm.

To characterize the contribution of each sequential digestion round to the total cell yield, cells from each of the eight fractions obtained from a single heart were seeded separately and monitored for 5 days (Figure 3a and Figure S1). Cell yield per fraction correlated positively with the degree of tissue digestion at collection: early and terminal fractions yielded low cell densities. In contrast, middle fractions (rounds 3–6) produced the largest number of spindle-shaped, actively proliferating fibroblasts. By Day 5, the middle fractions had formed confluent monolayers, whereas the terminal fractions showed substantially lower density (Figure 3a and Figure S1). To assess the dynamics of cell attachment and proliferation, we performed a time-course experiment using cells from pooled fractions, which we seeded into a single plate. Phase-contrast images showed that, at 24 h post-seeding, only a small number of cells had adhered, displaying a predominantly rounded morphology. By 72 h, fibroblasts exhibited a characteristic morphology, with evident cell expansion. The culture became confluent at 120 h, indicating high proliferative capacity and good viability of the isolated cells (Figure 3b).

### 2.4. Primary Cultures Maintained a Fibroblast-like Phenotype for up to 5 Days in Culture

**Figure 4:**
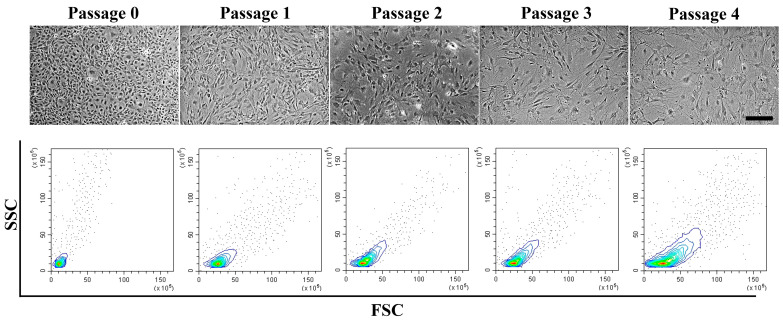
Serial passaging of cardiac fibroblasts isolated by Protocol 1. Representative phase-contrast images show cultures from passage 0 to passage 4. Flow cytometry contour plots of forward scatter and side scatter illustrate progressive hypertrophy and increased granularity with passage number. Scale bar: 250 μm.

Next, cardiac fibroblasts isolated by Protocol 1 were monitored across five passages (1:3 split ratio). At Passage 0, cultures displayed heterogeneous morphology with variable cell sizes. From Passage 1, morphology became uniformly spindle-shaped and consistent with cardiac fibroblast identity. This phenotype was maintained through Passage 2. From Passage 3 onward, a progressive increase in cell size and decrease in PDT were observed, consistent with a senescence-like phenotype (Figure 4 and Figure S3a). Flow cytometric analysis of forward scatter (FSC) and side scatter (SSC) confirmed increasing cell hypertrophy and granularity with successive passages (Figure 4). Consistently, quantification of mean FSC-A as a proxy for cell size, together with population doubling time, showed a significant increase at Passage 4 compared to Passage 1 (Figure S3a,b), further supporting this senescence-like phenotype. Western blot analysis showed detectable α-SMA expression across Passages 1–4. Although α-SMA levels tended to increase between Passages 1 and 3, these differences were not statistically significant, possibly due in part to the limited number of biological replicates (Figure S3c).

## 3. Discussion

Cardiac fibroblasts regulate myocardial homeostasis, extracellular matrix remodeling, scar formation, and adverse remodeling after cardiac injury. Their isolation from adult hearts remains challenging because the myocardial interstitium contains heterogeneous non-myocyte populations, including fibroblasts, smooth muscle cells, perivascular stromal cells, endothelial cells, and immune cells [2]. Moreover, no single marker identifies all cardiac fibroblast subsets across activation states or disease conditions [11]. Thus, isolation protocols must be adapted to the target cell population and downstream application. For cardiac fibroblast culture, the optimal method should preserve viability, limit contamination, and support fibroblast-enriched expansion.

Several isolation strategies have been reported. Classical enzymatic digestions are accessible because they require only standard dissection and culture equipment [6,7]. Other approaches use flow cytometry-based enrichment, reporter mice, selective adhesion, FACS, or magnetic separation to improve specificity [4,12,13,14,15]. Although these methods can increase purity, they require specialized infrastructure, longer processing times, and careful interpretation of marker combinations. We therefore focused on enzymatic strategies, which are practical, cost-effective, and suitable for routine primary cardiac fibroblast culture.

Here, we evaluated three enzymatic strategies that are commonly used to recover cardiac non-myocyte populations, either as the primary target or as a secondary fraction, to determine how effectively each approach supports the establishment and expansion of adult mouse cardiac fibroblast cultures. Although these approaches were developed for different primary purposes, all three can be used to obtain cardiac fibroblasts, and their relative performance in terms of viability, cellular composition, attachment, and expansion had not been directly assessed under the same experimental framework. Under these conditions, Protocol 1 provided the most favorable overall balance between viability, reduced endothelial and hematopoietic contamination, and expansion capacity. Similarly, under this predefined objective, sequential digestion with collagenase I and trypsin (Protocol 1) provided the most favorable balance between viability, fibroblast-enriched recovery, and expansion.

This advantage is likely related to the stepwise digestion strategy, which progressively releases interstitial cells across short digestion fractions and allows rapid neutralization of enzymatic activity. Although Protocol 1 recovered fewer Sca-1-positive cells immediately after isolation than the Liberase DH-based protocol, these cells showed better adherence and expansion, forming dense fibroblast-like cell monolayers. Another important advantage of Protocol 1 was the absence of detectable CD31-positive endothelial cells and CD45-positive hematopoietic cells in the freshly isolated fraction. This is relevant because contaminating endothelial or immune cells may affect culture purity, paracrine signaling, and downstream readouts. The fibroblast-enriched profile was confirmed after 5 days in culture, when passage 0 adherent cells expressed S100A4, PDGFR-alpha, and α-SMA, while remaining negative for CD31 and CD45. Protocol 3, based on Liberase DH digestion, recovered the broadest non-myocyte profile, including the highest proportions of Sca-1-positive and CD31-positive cells. This makes it useful for immediate flow cytometric profiling, cell sorting, or single-cell transcriptomic applications, but less suitable for expanding fibroblast-enriched cultures. Protocol 2 remains useful when cardiomyocyte and non-myocyte fractions must be obtained from the same heart. However, the higher mortality and weaker expansion after retrograde perfusion limit its use as a dedicated fibroblast culture protocol.

Bulk RNA sequencing analysis provided additional support for the predominant fibroblast identity of the cultured cells isolated using Protocol 1. Seven of the 10 most highly expressed genes that could be assigned to a cardiac cell population were fibroblast-associated, including Col1a1, Col1a2, Col3a1, Bgn, and Fstl1. Consistently, canonical fibroblast markers, including Pdgfra, Tcf21, Dcn, Lum, and fibrillar collagens, showed higher overall expression than cardiomyocyte-associated genes. The use of a multi-gene signature derived from the Tabula Muris Senis atlas was considered more informative than reliance on a single marker, given the recognized transcriptional heterogeneity of cardiac fibroblasts [10,16,17]. The expression of Acta2 and other smooth muscle/mural-associated genes may reflect the acquisition of a contractile, myofibroblast-like phenotype during culture, as conventional rigid culture substrates are known to promote basal cardiac fibroblast activation and α-SMA expression [18,19]. These transcriptomic findings complement the morphological and flow-cytometric data. However, they were interpreted descriptively rather than as quantitative estimates of cellular composition, and a minor contribution from residual mesenchymal or mural populations cannot be entirely ruled out.

Our results also highlight the importance of limiting the number of passages. Replicative senescence following serial passaging is a well-documented phenomenon in primary cardiac fibroblast cultures [20], reflecting their marked phenotypic plasticity. Accordingly, prolonged culture and repeated passaging may promote phenotypic drift, increased cell size, reduced proliferative capacity, and myofibroblast-like activation [21,22]. Consistent with these changes, population doubling time increased significantly at Passage 4, confirming the progressive decline in proliferative capacity observed at later passages. α-SMA protein was detectable across Passages 1–4 and showed a tendency to increase from Passage 1 to Passage 3. Together, these findings support the preferential use of cardiac fibroblasts up to Passage 2 to preserve proliferative capacity and phenotypic consistency.

The main limitation is that the comparison between protocols was based on viability, flow cytometric characterization, culture performance, and bulk RNA-sequencing data analysis rather than single-cell transcriptomic validation or dedicated functional assays of fibroblast responsiveness. These aspects should be further investigated in future studies to more comprehensively define the cellular composition, activation state, and functional properties of the isolated cells. However, cardiac fibroblasts isolated using the sequential digestion protocol described here have been previously employed in next-generation sequencing studies in our laboratory [23], where their phenotypic purity was confirmed by flow cytometry (PDGFRα^+^, CD31^−^, CD45^−^), immunofluorescence staining for collagen I, collagen III, and α-SMA, and metabolic profiling, thereby providing additional evidence for the reliability of this isolation approach.

Taken together, these findings identify the sequential collagenase I/trypsin digestion protocol as the most suitable strategy for routine primary adult mouse cardiac fibroblast culture. While Liberase DH digestion is preferable for broad recovery of freshly isolated non-myocyte populations, Protocol 1 provides the best balance between viability, reduced contamination, and robust expansion of fibroblast-like cells for downstream functional and molecular studies.

## 4. Materials and Methods

### 4.1. Animals

C57BL/6J mice were purchased from The Jackson Laboratory (Bar Harbor, ME, USA) and bred under controlled conditions, with standard housing (12 h light/dark cycle, 21 °C, 55–60% humidity) and ad libitum access to chow and water. The mouse cohort used in this study comprised 40 male and female mice, aged 2–4 months and weighing 18–23 ± 2 g. Anesthesia was induced by intraperitoneal injection of ketamine/xylazine at final doses of 100 mg/kg ketamine (CP-Pharma Handelsgesellschaft mbH, Burgdorf, Germany) and 10 mg/kg xylazine (Bioveta a.s, Ivanovice na Hane, Czech Republic). Following anesthesia, mice were euthanized by heart excision. All animal procedures were approved by the institutional ethics committee and conducted in accordance with national regulations governing the use of laboratory animals.

### 4.2. Reagents, Buffers, and Culture Medium

All buffers were prepared using ultrapure water (18.2 MΩ · cm), sterile-filtered through 0.22 um filters (Merck KGaA, Darmstadt, Germany), and stored at 4 °C for up to two weeks unless otherwise specified. Fresh digestion buffers were prepared on the day of isolation.

HBSS buffer was prepared from 100× stock solutions containing NaCl/KCl, Na_2_HPO_4_/KH_2_PO_4_, CaCl_2_.2H_2_O, MgSO_4_.7H_2_O, and NaHCO_3_ (Sigma-Aldrich, St. Louis, MO, USA). (See Tables S1 and S2 for complete composition).

Cardiac fibroblast culture medium (CFCM) consisted of DMEM/F12 (Sigma-Aldrich, St. Louis, MO, USA) supplemented with 10% fetal bovine serum (Gibco, Thermo Fisher Scientific, Waltham, MA, USA), 1% penicillin/streptomycin (Gibco, Thermo Fisher Scientific, Waltham, MA, USA), 2 mM L-glutamine (Gibco, Thermo Fisher Scientific, Waltham, MA, USA), 0.5 mM sodium pyruvate (Sigma-Aldrich, St. Louis, MO, USA), 14.64 mM NaHCO_3_ (Sigma-Aldrich, St. Louis, MO, USA), 10 ng/mL fibroblast growth factor-beta (R&D Systems, Minneapolis, MN, USA) and 10 μg/mL insulin (Sigma-Aldrich, St. Louis, MO, USA). FGF-beta and insulin were added immediately before use (Table S3). Following isolation, cells obtained using all three protocols were cultured under identical conditions using the same CFCM formulation.

Protocol 1 digestion buffer contained 100 ug/mL collagenase type I (Sigma-Aldrich, St. Louis, MO, USA), 0.1% trypsin (Sigma-Aldrich, St. Louis, MO, USA), and 1×HBSS, freshly used (see Table S4 for complete composition).

Protocol 2 digestion buffer contained collagenase type II (Gibco, Thermo Fisher Scientific, Waltham, MA, USA) at 1 mg/mL in perfusion buffer, prepared fresh for retrograde perfusion (see Table S5 for complete composition).

Protocol 3 digestion buffer contained 200 ug/mL Liberase DH (Sigma-Aldrich, St. Louis, MO, USA) in 1X HBSS (see Table S6 for complete composition).

### 4.3. Protocol 1: Sequential Enzymatic Digestion with Collagenase I and Trypsin

**Scheme 1:**
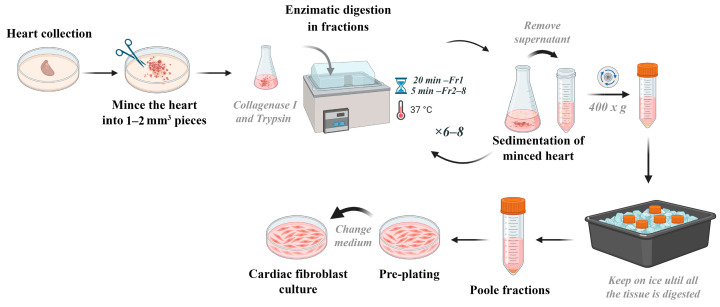
Schematic overview of Protocol 1. Following cardiac excision, the heart is minced into approximately 1 mm fragments and subjected to sequential enzymatic digestions with collagenase I and trypsin at 37 °C. Supernatants are collected after each digestion round, pooled, centrifuged at 400× *g*, and resuspended in cardiac fibroblast culture medium (created in BioRender. Rusu, E. G. (21 July 2026) https://BioRender.com/inw1gka).

Protocol 1 was designed as a Langendorff-free cardiac fibroblast isolation method adapted from a previously described approach [7]. This method served as a reference for the other protocols (Scheme 1).

Following deep anesthesia, the thoracic cavity was opened, and the heart was excised and transferred into ice-cold sterile PBS. All subsequent steps were performed under sterile conditions in a cell culture hood. The heart was washed in a Petri dish with ice-cold PBS and gently compressed with forceps to remove residual blood. The tissue was next minced using straight and curved scissors into 1 mm fragments (see Appendix A), then transferred from the ice-cold Petri dish into a 50 mL Erlenmeyer flask containing 10 mL of digestion buffer, and incubated at 37 °C under constant stirring for 20 min. After the first digestion round, the suspension was gently triturated by pipetting ten times using a 5 mL pipette. Tissue fragments were allowed to settle for 1 min (see Appendix A), and the supernatant was collected into a new 50 mL tube containing cold 10% FBS to inactivate the enzymes. Subsequent digestion rounds were performed using 8 mL of digestion buffer for 5 min each at 37 °C under constant stirring. After each round, the suspension was gently triturated, the tissue was allowed to sediment, and the cell-containing supernatant was collected into cold FBS. From digestion fractions 3–4 onward, tissue fragments were gently mechanically dissociated using needles of decreasing diameter once the tissue had become pale, soft, and friable. Tissue fragments were not forced through needles; excessive pressure was avoided to minimize cell damage. Collected fractions were pooled, centrifuged at 400× *g* for 5 min at 4 °C, and resuspended in CFCM. All cells obtained were seeded into 100 mm culture dishes and maintained at 37 °C in 5% CO_2_. After 24 h, the medium was removed, cultures were washed three times with warm PBS, and fresh pre-warmed CFCM was added. On day 5, confluent cultures were washed with PBS and detached using pre-warmed trypsin-EDTA for 2–5 min at 37 °C. Trypsin was neutralized with cold CFCM, and cells were sub-cultured at a 1:3 split ratio (see Appendix A). All the equipment needed for this protocol is described in detail in the Supplemental Files, Tables S11 and S12.

### 4.4. Protocol 2: Retrograde Coronary Perfusion with Collagenase II

**Scheme 2:**
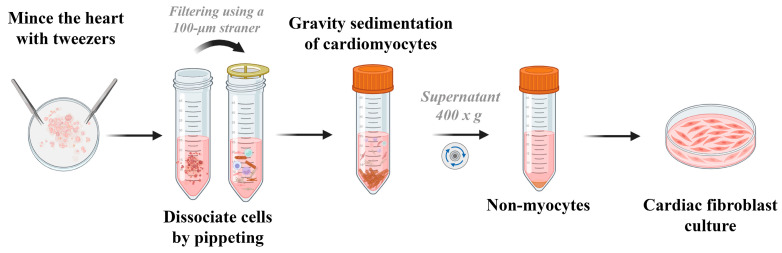
Schematic overview of Protocol 2. Following retrograde coronary perfusion and tissue dissociation, cardiomyocytes are separated from non-myocyte cells by gravity sedimentation. The non-myocyte supernatant is centrifuged to obtain a cell pellet used for cardiac fibroblast culture (created in BioRender. Rusu, E. G. (21 July 2026) https://BioRender.com/k8h4zlp).

Protocol 2, based on retrograde coronary perfusion with collagenase II, was selected when both cardiomyocytes and non-myocyte cells were of interest for recovery from a single heart. In this workflow, the fibroblast-enriched non-myocyte fraction was obtained from the supernatant remaining after gravity sedimentation of cardiomyocytes (Scheme 2). Before organ excision, the perfusion system was assembled and equilibrated. The tubing was connected to the peristaltic pump (P-1; Cytiva, Marlborough, MA, USA); all lines were primed with EDTA buffer pre-warmed to 37 °C to avoid air bubbles, and the water bath (WNB/WNE/WPE; Memmert GmbH + Co. KG, Schwabach, Germany) was set to 37 °C to maintain a constant temperature throughout the perfusion procedure (Scheme S1). After anesthesia, heparin (Sigma-Aldrich, St. Louis, MO, USA) was administered intraperitoneally at 100 U/mL to prevent blood coagulation. The thoracic cavity was opened, the heart was exposed, and the aorta was carefully cut at the level of the aortic arch, leaving approximately 0.5 cm of free aorta for cannulation. The excised heart was immediately transferred to a Petri dish containing 5–10 mL EDTA buffer. The aorta was then cannulated with a blunt 20G needle prefilled with EDTA buffer, taking care to avoid introducing air bubbles into the coronary circulation (Scheme S2). Successful cannulation was indicated by rapid clearing of blood from the coronary vessels and by the appearance of transparent vessels during perfusion.

Hearts were perfused at 37 °C and 2.5 mL/min using the following sequence: 3 min with EDTA buffer (Table S7), 3 min with perfusion buffer (Table S8), and 8–15 min with digestion buffer containing collagenase II. Digestion was considered complete when the heart became visibly pale, softened, and flaccid, indicating efficient loosening of the myocardial tissue. After digestion, the heart was removed from the cannula and transferred to STOP buffer (Table S9). Tissue dissociation was completed under sterile conditions using curved forceps and gentle pipetting. The resulting cell suspension was filtered through a 100 μm cell strainer, and cardiomyocytes were separated from the non-myocyte fraction by gravity sedimentation for 20 min at room temperature. The supernatant containing non-myocyte cells was collected and centrifuged at 400× *g* for 5 min at 4 °C. The resulting cell pellet was resuspended in cardiac fibroblast culture medium and plated as described for Protocol 1. The equipment used in this protocol is listed in the Supplemental Files, Tables S11 and S12.

### 4.5. Protocol 3: Continuous Enzymatic Digestion with Liberase DH

**Scheme 3:**
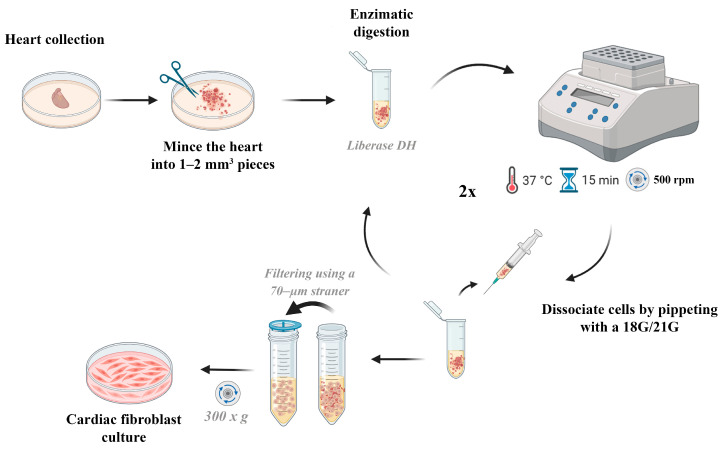
Schematic overview of Protocol 3. Minced cardiac tissue undergoes two sequential 15 min enzymatic digestion rounds with Liberase DH at 37 °C, with mechanical dissociation between rounds. Cells are filtered through a 70 μm strainer and centrifuged before seeding or flow cytometric analysis (created in BioRender. Rusu, E. G. (21 July 2026) https://BioRender.com/fbgr38j).

Protocol 3 used Liberase DH (Sigma-Aldrich, St. Louis, MO, USA) to enable rapid, broad dissociation of the cardiac non-myocyte compartment. This method was chosen for evaluation based on the hypothesis that enzyme blends containing collagenase and neutral protease activity may increase the recovery of interstitial and vascular cell populations (Scheme 3).

Hearts were prepared as described for Protocol 1, but tissue fragments were cut to approximately 2–3 mm and transferred into digestion buffers containing Liberase DH (Sigma-Aldrich, St. Louis, MO, USA). Digestion was performed in a thermomixer at 37 °C for 15 min at 500 rpm, followed by gentle mechanical dissociation through an 18G needle (Changzhou Shuangma Medical Devices Co., Ltd., Changzhou, Jiangsu, China) A second 15 min digestion was then performed under the same conditions, followed by dissociation through a 21G needle (Changzhou Shuangma Medical Devices Co., Ltd., Changzhou, Jiangsu, China).

Digestion was stopped by adding cold PBS containing 10% FBS. The suspension was filtered through a 70 μm strainer (Corning Incorporated, Corning, NY, USA) and centrifuged at 400× *g* for 5 min at 4 °C. The pellet was resuspended in CFCM and plated using the same culture conditions as Protocol 1. The equipment used in this protocol is described in the Supplemental Files, Tables S11 and S12.

### 4.6. Flow Cytometry

Flow cytometry was used to characterize cells immediately after isolation (fresh cell analysis) and after 5 days in culture (cultured cells). For day 5 analysis, adherent cells were detached with 0.25% trypsin for 2 min at 37 °C and centrifuged at 400× *g* for 5 min at 4 °C. Cell pellets were resuspended at 5 × 10^5^ cells/mL in PBS containing 5% species-matched blocking serum and incubated for 30 min at 4 °C to block Fc receptors and reduce non-specific binding. Cells were then incubated with fluorochrome-conjugated antibodies against Sca-1, PDGFR-alpha, CD31, and CD45 (Additional details are provided in Table S10) for 40 min on ice, protected from light. After staining, cells were washed once with 1 mL 1× PBS and centrifuged at 400× *g* for 5 min at 4 °C. The final cell pellet was resuspended in 1× PBS supplemented with 2% FBS and 2 μg/mL propidium iodide (PI). Samples were acquired on a CytoFLEX flow cytometer (Beckman Colter Life Sciences, Brea, CA, USA), and a minimum of 20,000 events were recorded per sample. Viable cells were identified based on PI exclusion, and data were analyzed using the appropriate gating strategy and isotype controls. Data were analyzed with CytExpert software (version 2.1; Beckman Colter Life Sciences, Brea, CA, USA).

For intracellular antigen staining, the same standard staining protocol was used, except that cells were fixed and permeabilized before the blocking step. Briefly, cells were fixed with 4% PFA (Sigma-Aldrich, St. Louis, MO, USA) for 15 min at room temperature, washed with 1× PBS, and permeabilized with 0.7% Tween-20 (Sigma-Aldrich, St. Louis, MO, USA) for 15 min. After washing, cells were blocked in PBS supplemented with 5% species-matched serum and then incubated with the primary S100A4 and anti-α-SMA antibody (Additional details are provided in Table S10), followed by the appropriate fluorochrome-conjugated secondary antibody. After final washing, cells were resuspended in PBS containing 2% FBS and analyzed by flow cytometry as described above.

### 4.7. Bulk RNA-Sequencing-Based Assessment of Cardiac Fibroblast Identity

The transcriptional identity of cultured cardiac fibroblasts was evaluated using our bulk RNA-sequencing data (GSE279927) from three biological samples and cell-type-specific marker genes derived from the mouse heart Smart-seq2 dataset of the Tabula Muris Senis atlas. Cell-type-specific genes were identified in Scanpy (version 1.11.5) using the Wilcoxon rank-sum test (scanpy.tl.rank_genes_groups), comparing each cardiac cell population (fibroblast, endothelial, cardiomyocyte, immune, smooth muscle/mural) against all remaining cardiac cell populations. For genes associated with more than one population, the assignment with the highest specificity score was retained; log fold-change was used as a tiebreaker in cases of equal specificity scores. The specificity score was calculated as specificity score = (pct_nz_group − pct_nz_reference) × log fold change. The 10 most highly expressed bulk RNA-sequencing genes that could be assigned to one of the five cell-type categories were selected based on their average normalized expression value; genes without a cell-type assignment were excluded from this analysis.

A complementary canonical marker panel, comprising established fibroblast, endothelial, cardiomyocyte, immune, and smooth muscle/mural markers, was also analyzed. Bulk RNA-sequencing counts were normalized using DESeq2 (performed by Novogene (UK) Company Limited, (Cambridge, UK). and transformed as log2 (normalized expression + 1). For each gene, mean expression and standard deviation were calculated across the three biological samples, and the median expression was determined within each marker category. Data processing and visualization were performed in R (version 4.6.0).

### 4.8. Statistical Analysis

Statistical analyses were performed using GraphPad Prism version 11.0.2. Data are presented as mean ± standard deviation (SD). Comparisons among groups were conducted using one-way or two-way analysis of variance (ANOVA), as appropriate. When applicable, the post hoc multiple-comparison tests used are specified in the corresponding figure legends. Differences were considered statistically significant at *p* < 0.05.

## 5. Conclusions

In conclusion, this study provides a comparative evaluation of three enzymatic strategies for isolating cardiac fibroblast-enriched cell populations from adult mouse hearts. Among the tested approaches, the sequential digestion protocol based on collagenase I and trypsin emerged as the most suitable method for establishing primary cardiac fibroblast cultures. Protocol 1 combined low post-isolation cell death, minimal contamination from endothelial and hematopoietic cells, efficient adherence, and robust expansion of fibroblast-like cells in culture. Flow cytometric characterization further confirmed the fibroblast-enriched phenotype of passage 0 cultures, with expression of S100A4, PDGFRα, and α-SMA and absence of detectable CD31^+^ and CD45^+^ contaminating cells. Although Liberase DH-based digestion provided broader recovery of freshly isolated cardiac non-myocyte populations, including endothelial cells, Protocol 1 offered the most favorable balance between viability, purity, and culture performance. Therefore, the sequential digestion protocol may provide a robust experimental platform for advancing mechanistic studies of cardiac fibroblast biology in health and disease.

## Data Availability

The original contributions presented in this study are included in the article/Supplementary Materials. Further inquiries can be directed to the corresponding author.

## Supplementary Materials

The following supporting information can be downloaded at: https://www.mdpi.com/article/10.3390/ijms27156630/s1.

## Abbreviations

The following abbreviations are used in this manuscript:

cFb	cardiac fibroblast
ECM	extracellular matrix
CFCM	cardiac fibroblast culture medium
FACS	fluorescence-activated cell sorting
FBS	fetal bovine serum
HBSS	Hanks’ balanced salt solution
PBS	phosphate-buffered saline
α-SMA	alpha-smooth muscle actin
PDGFR-alpha	platelet-derived growth factor receptor alpha
PI	propidium iodide

## Appendix A. Technical Notes

Tissue fragments should be approximately 1 mm for Protocol 1. Larger fragments digest incompletely, whereas very small fragments may be overdigested or lost.

Only the cell-containing supernatant should be collected during Protocol 1. Floating tissue fragments should not be aspirated.

The 24 h plating step is important for removing debris and reducing non-adherent contaminating cells.

Fibroblasts should not be passaged before reaching at least 80% confluence.

Primary cardiac fibroblasts are recommended for experimental use up to passage 2.

Propidium iodide should be added immediately before flow cytometric acquisition to avoid increased background staining.
