# Supplementary material for: Establishing Primary Cultures of Adult Mouse Cardiac Fibroblasts: A Comparative Evaluation of Three Experimental Protocols
Source: Int J Mol Sci. 2026 Jul 25;27(15):6630. doi: 10.3390/ijms27156630 (PMC13465877; doi:10.3390/ijms27156630)
Supplement: Supplementary file 1 [file ijms-27-06630-s001.zip › ijms-4431415-supplementary.pdf]

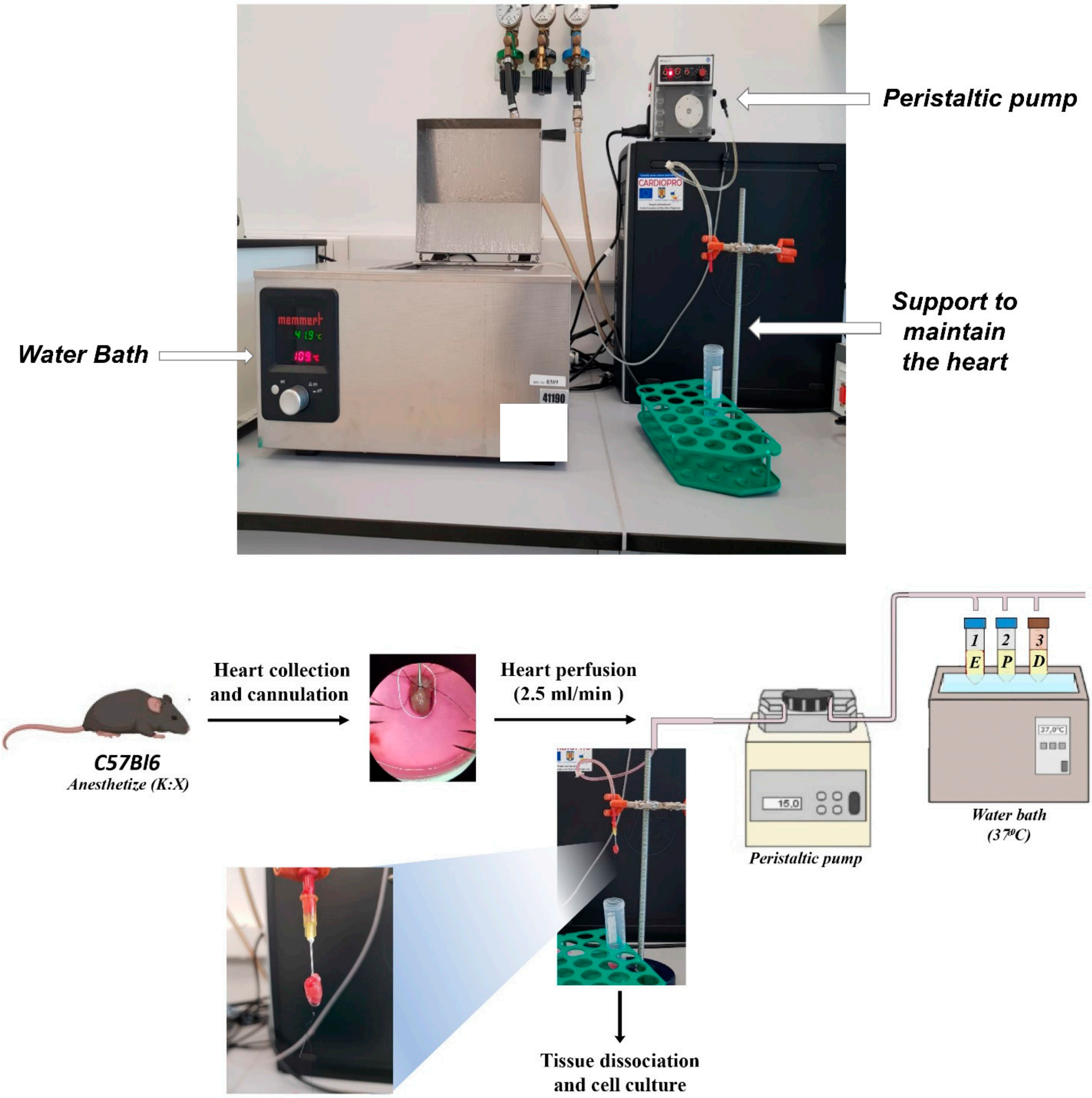

Scheme S1. Components of the isolation system. The system comprises a water bath set at 37°C, a peristaltic pump set at 2.5 mL/min, sterile silicone tubing, and a support stand maintaining the cannulated heart connected to the perfusion circuit.

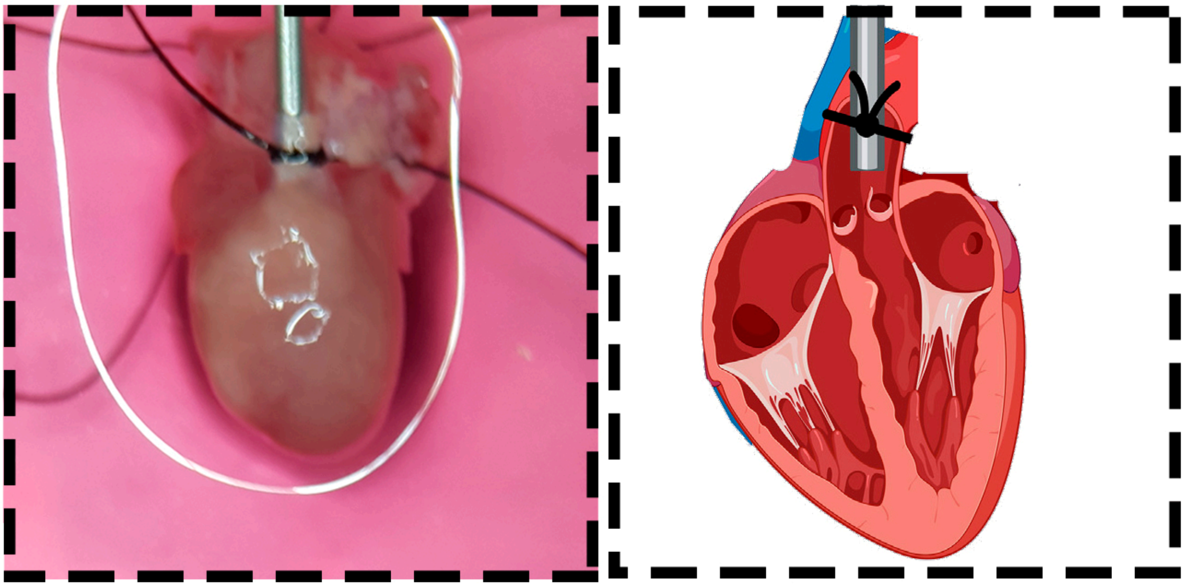

Scheme S2. Cannulation of the mouse heart for retrograde perfusion. Left — excised adult mouse heart cannulated and connected to the perfusion circuit. Right — schematic illustration of cannula positioning within the aortic lumen relative to the aortic valve and suture ligature

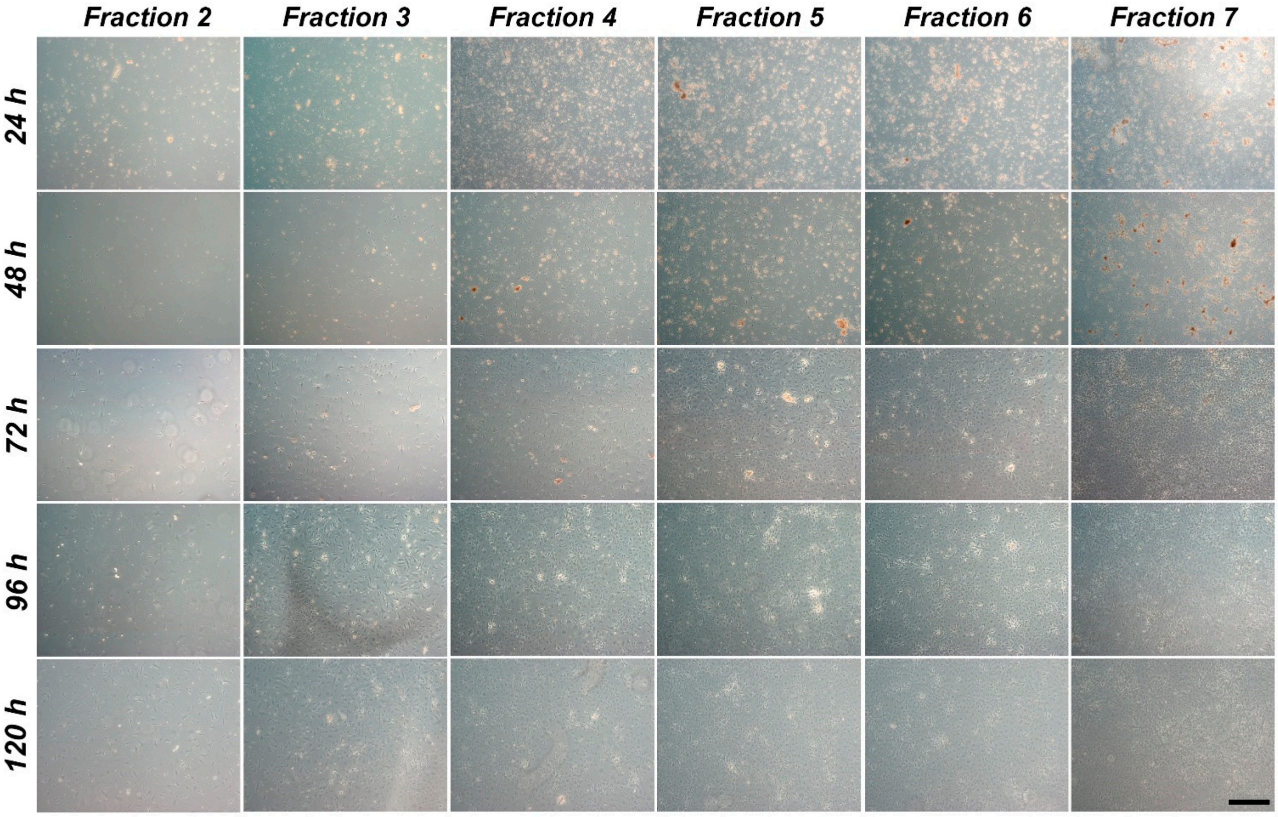

Figure S1. Fraction-resolved analysis of cardiac fibroblast expansion following sequential enzymatic digestion. Representative phase-contrast images showing adherent cardiac fibroblast cultures obtained from individual digestion fractions collected during Protocol 1. Fractions 2–7 were seeded separately and monitored over time at 24, 48, 72, 96, and 120 h after isolation. Fractions 1 and 8 were not included in the montage for image clarity, as they contained only a low number of adherent cells. Scale bar: 500 μm

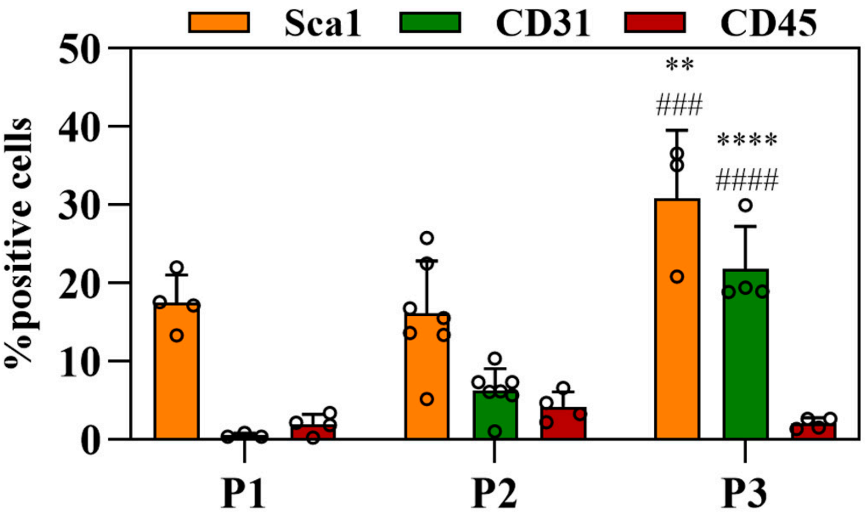

Figure S2. Quantitative comparison of freshly isolated cell populations across the three isolation protocols. Quantification of Sca-1-, CD31-, and CD45-positive cells immediately after isolation using Protocols 1–3. Data are presented as mean ± SD, with individual biological replicates shown. Statistical analysis was performed using two-way ANOVA followed by Tukey's multiple-comparisons test. **$p < 0.01$, and ****$p < 0.0001$ versus Protocol 1; ###$p < 0.001$, and ####$p < 0.0001$ versus Protocol 2.

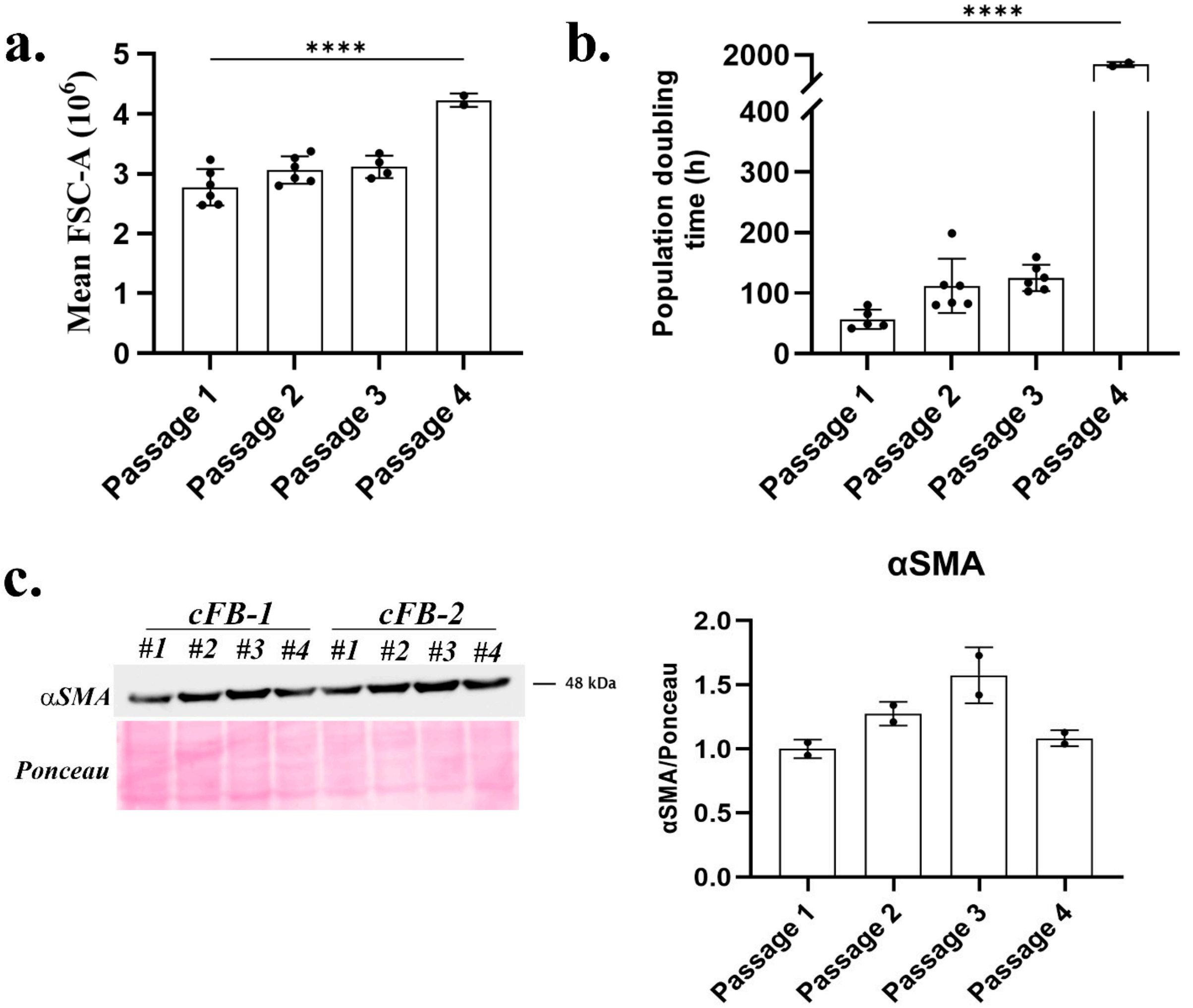

Figure S3. Cellular senescence markers of cardiac fibroblasts across serial passages. (a) Mean forward scatter area (FSC-A), used as an indirect measure of cell size, measured by flow cytometry at passages 1–4. (b) Population doubling time (h) at passages 1–4; Both cell size and doubling time increased markedly at passage 4, consistent with the onset of replicative senescence. Data are presented as mean ± SD (n = 2–6 per group); Statistical analysis was performed by ordinary one-way ANOVA with Dunnett's multiple comparisons test; **** $p < 0.0001$.(c) Representative Western blot and densitometric quantification of α-SMA protein expression across Passages 1–4. α-SMA signal was normalized to total protein staining using Ponceau S. Data are presented as mean ± SD; n = 2 independent biological replicates. Because the assumption of equal variances was not met, differences among passages were analyzed using Brown–Forsythe and Welch's one-way ANOVA tests. No statistically significant differences were detected among passages (Brown–Forsythe ANOVA: F* = 7.989, p = 0.1321; Welch's ANOVA: W = 4.213, p = 0.1862).

Reagents for Cardiac Fibroblast Isolation

All buffers were prepared in ultrapure 18.2 MΩ·cm H_2O, sterile-filtered (0.22 µm), and stored at 4°C for up to two weeks unless stated otherwise.

Table S1. HBSS Buffer (1×)

Stock	Compound	Amount	Solvent (mL)
Stock 1	NaCl / KCl	8.0 g / 0.40 g	100
Stock 2	Na_2HPO_4 / KH_2PO_4	0.35 g / 0.60 g	100
Stock 3	$CaCl_2 \cdot 2H_2O$	0.95 g	50
Stock 4	$MgSO_4 \cdot 7H_2O$	1.23 g	50
Stock 5	$NaHCO_3$	0.35 g	10

Table S2. HBSS final assembly (100 mL total):

Component	Volume (mL)
Stock 1	10
Stock 2	1
Stock 3	1
Distilled H_2O	86
Stock 4	1
Stock 5	1

Final composition: 0.137 M NaCl, 5.4 mM KCl, 0.25 mM Na_2HPO_4, 0.44 mM KH_2PO_4, 1.3 mM $CaCl_2$, 1 mM $MgSO_4$, 4.2 mM $NaHCO_3$.

Table S3. Cardiac Fibroblast Culture Medium

Compound	Final concentration
DMEM/F12 (Sigma, D8062)	—
Fetal Bovine Serum (FBS)	10%
Penicillin/Streptomycin	1%
L-Glutamine	2 mM (1×)
Sodium pyruvate	0.5 mM
$NaHCO_3$#	29.02 mM
Fibroblast Growth Factor (FGF-β)	10 ng/mL*
Insulin	10 µg/mL*

**FGF-β and insulin are added fresh immediately before use.*

# $NaHCO_3$ was supplemented only when its concentration in the basal medium was lower than 29.02 mM, to achieve a final concentration of 29.02 mM.

Table S4. Digestion Buffer: Protocol 1

Component	Stock	Final conc.

Collagenase type I	10 mg/mL	100 µg/mL
Trypsin	0.25%	0.1%
HBSS buffer	1×	1×

Table S5. Digestion Buffer: Protocol 2

Component	Stock	Final conc.	Amount for 50 mL
Collagenase type II (Gibco)	290 U/mg	1 mg/mL	50 mg
Perfusion Buffer (pH 7.8)	—	—	50 mL

Table S6 Digestion Buffer: Protocol 3

Component	Final conc.
Liberase DH (Roche)	200 µg/mL
HBSS (1×)	—

Table S7. ***EDTA Buffer***

Compound	MW (g/mol)	Final conc. (mmol/L)	g / L	Notes
NaCl	58.44	130	7.597	
KCl	74.55	5	0.373	
$NaH_2PO_4 \cdot H_2O$	138.0	0.5	0.069	
HEPES	238.3	10	2.383	
Glucose	180.16	10	1.802	
BDM (2,3-butanedione monoxime)	101.1	10	1.011	Inhibits cardiac contractions
Taurine	125.15	10	1.252	
$EDTA \cdot 2H_2O$	372.24	5	1.861	Ca^{2+} chelator

Prepare in 1 L ultrapure H_2O. Adjust pH to 7.8 with NaOH. Sterile-filter (0.22 µm). Store at 4°C for up to one week..

Table S8. ***Perfusion Buffer***

Compound	MW (g/mol)	Final conc. (mmol/L)	g / L	Notes
NaCl	58.44	130	7.597	
KCl	74.55	5	0.373	
$NaH_2PO_4 \cdot H_2O$	138.0	0.5	0.069	
HEPES	238.3	10	2.383	
Glucose	180.16	10	1.802	
BDM (2,3-butanedione monoxime)	101.1	10	1.011	Inhibits cardiac contractions
Taurine	125.15	10	1.252	

$MgCl_2 \cdot 6H_2O$	203.30	1	0.203	Replace EDTA with $MgCl_2$

Identical to EDTA Buffer except $EDTA \cdot 2H_2O$ is replaced by 1 mM $MgCl_2 \cdot 6H_2O$. Adjust pH to 7.8 with NaOH. Sterile-filter (0.22 µm). Store at 4°C for up to one week.

Table S9. *STOP Buffer*

Component	Final conc.	Volume (per 10 mL)
Perfusion Buffer	—	9.5 mL
Fetal Bovine Serum (FBS)	5%	0.5 mL

Prepare fresh on day of isolation.

Table S10. Flow Cytometry Panel

Target	Cat. No. / Clone	Fluorochrome	Host	Dilution	Supplier	Purpose / Panel
Sca-1 (Ly6A/E)	108108	PE	Rat	1:50	BioLegend	Mesenchymal / cFb identification
PDGFRα	135916	BV605	Rat	1:50	BioLegend	Fibroblast identity (culture)
α-SMA (1° Ab)<br><br>Goat a-Rabbit igG AF700 (2° Ab)	ab5694<br><br>A-21038	-<br><br>2°: AF700)	Rabbit	1°: 1:20;<br><br>2°: 1:200	Abcam<br><br>Invitrogen	Myofibroblast marker (intracellular);<br>Secondary antibody
S100A4	NBP2-54400F	FITC	Mouse	1:33	Novusbio	Mesenchymal / cFb identification
CD31 (PECAM-1)	102414	AF488	Rat	1:33	BioLegend	Endothelial cell marker
CD45	109814	APC	Mouse	1:80	BioLegend	Hematopoietic cell marker
CD45	103126	PB	Rat	1:200	BioLegend	Hematopoietic cell marker
Propidium Iodide	P4170	PI	—	1:100	Sigma	Viability exclusion

Immunophenotypic characterization was performed using the following antibodies:

Table S11. Surgical Equipment

Item	Specification / Notes	Quantity
Skin forceps	Standard tissue forceps for initial skin incision	1
Blunt-end scissors	For opening the thoracic cavity without damaging underlying organs	1
Curved-end forceps	For manipulation of the heart and aorta during excision	1
Sharp-end scissors	For cutting at the level of the aortic arch; leave ~0.5 cm of aorta free	1
Reynolds full-curved hemostatic forceps	For clamping the heart immediately after exposure; minimises warm ischemia time	1
Straight-end forceps	For general tissue handling and cannulation support	1

Blunt 20G needle (cannula)*	Pre-filled with EDTA Buffer; used for aortic cannulation (Section 4.4.1).	1
Surgical suture*	For securing the cannula in the aortic lumen	As needed

Table S12. Isolation and Culture Equipment

Item	Specification / Notes	Quantity
Curved-tip scissors	For mincing the heart; sterilize before use	1
Sharp-tip scissors	For mincing the heart; sterilize before use	1
Petri dish, 60 mm (non-adherent)	Used for heart washing, mincing, and cannulation steps	As needed
Micropipettes (P20, P200, P1000)	For reagent addition, supernatant collection, and cell transfer	1 set
Serological pipettes (5 mL, 10 mL, 25 mL)	For buffer addition and cell suspension transfer	As needed
Falcon tubes, 50 mL	For collecting cell suspensions and centrifugation	5–10 per isolation
Falcon tubes, 15 mL	For calcium re-introduction steps (cardiomyocyte protocol)	2–4 per isolation
Microcentrifuge tubes, 1.5 mL and 2 mL	For Liberase DH digestion (Protocol 3)	As needed
Sterile aluminium foil	For covering the Erlenmeyer flask during enzymatic digestion (Protocol 1)	As needed
Sterile filter, 0.22 µm	For sterile-filtering digestion buffers and culture media	1 per buffer/medium
Cell strainer, 100 µm	For filtering cell suspension after digestion (Protocols 2 and CM isolation)	1 per isolation
Cell strainer, 70 µm	For filtering cell suspension after Liberase DH digestion (Protocol 3)	1 per isolation
Erlenmeyer flask, 50 mL (autoclaved)	Used for sequential digestion under constant agitation (Protocol 1)	1 per isolation
Water bath, 37°C*	For maintaining buffer temperature during retrograde perfusion	1
Peristaltic pump*	Set to 2.5 mL/min; used for retrograde coronary perfusion (Protocols 2 and CM isolation)	1
Thermomixer	Set to 37°C, 500 RPM; used for Liberase DH digestion (Protocol 3)	1
Centrifuge (refrigerated)	For cell pelleting at 400 × g, 4°C	1
Biosafety cabinet (Class II)	All post-excision steps are performed under sterile conditions	1
CO_2 incubator	37°C, 5% CO_2; for cell culture after seeding	1
Hemocytometer or automated cell counter	For counting cardiomyocytes before seeding at 25,000 cells/mL	1
Syringes with needles (18G, 20G, 21G, 23G)	For mechanical dissociation during Protocols 1 and 3, the gauge sequence depends on the protocol	As needed

Tissue culture dishes, 100 mm	For primary cFb seeding	1 per isolation
Tissue culture plates, 6-well	For fraction-resolved analysis of Protocol 1 (one well per fraction)	As needed
Laminin-coated surfaces	5 µg/mL laminin in PBS, pre-coated for 1 h at 37°C; required for cardiomyocyte adhesion	As needed
